# A Plant‐Diversity Dark Spot at the Intersection of Three Biodiversity Hotspots: Environmental Drivers of Brassicaceae Richness in Türkiye

**DOI:** 10.1002/ece3.73246

**Published:** 2026-03-16

**Authors:** İlayda Dumlupınar, Barış Özüdoğru, Hakan Gür

**Affiliations:** ^1^ Graduate School of Science and Engineering Hacettepe University Ankara Türkiye; ^2^ Department of Biology, Faculty of Science Hacettepe University Ankara Türkiye; ^3^ Anatolian Biogeography Research Laboratory Kırşehir Ahi Evran University Kırşehir Türkiye

**Keywords:** Anatolia, aridity, Brassicaceae, climatic stability, endemism, Neotethys closure, spatial regression models, species richness, tectonic uplift, topographic complexity

## Abstract

We provide the first nationwide, spatially explicit analytical framework for assessing how environmental and spatial processes structure species and endemic richness in Brassicaceae across Türkiye, a global plant‐diversity “dark spot.” We compiled an updated national checklist and > 15,000 unique, georeferenced herbarium records for Brassicaceae and aggregated them to 0.5° × 0.5° grid cells. We quantified species and endemic richness per cell and related them to contemporary climate, aridity, long‐term climatic stability, net primary productivity, human modification, and topography. We compared ordinary least squares with spatial regression models, performed variation partitioning to separate environmental and spatial components, and applied geographically weighted regression to assess spatial variation in relationships. In doing so, we found that species and endemic richness peak in southern Anatolia (the Western and Central Taurus Mountains) and along the Anatolian Diagonal, with additional species‐rich areas in the northern parts of the Central Anatolian Plateau. For species richness, topographic roughness has a strong positive effect, whereas annual precipitation has a strong negative effect, consistent with concentration in topographically heterogeneous, relatively dry landscapes. For endemic richness, elevation and roughness have strong positive effects, whereas temperature, past‐climatic stability index, and aridity index have strong negative effects, indicating that endemics are concentrated in high‐elevation, topographically heterogeneous, cooler, climatically stable, and relatively dry landscapes. Environmental gradients explain substantially more variation than purely spatial structure, and geographically weighted regression shows that model fit is highest in the Central Taurus Mountains. Overall, topographically complex, climatically stable mountain systems in southern Anatolia and along the Anatolian Diagonal simultaneously act as centers of species and endemic richness in Brassicaceae. These regions emerge as key conservation priorities for safeguarding both current diversity and evolutionary potential in one of the world's plant‐diversity “dark spots”.

## Graphical abstract

Türkiye lies at the intersection of three global biodiversity hotspots yet is recognised as a global plant‐diversity "dark spot." Using > 15,000 georeferenced herbarium records and spatial regression models, we examined the environmental drivers of Brassicaceae species and endemic richness across the country. Species richness increased with topographic roughness and decreased with annual precipitation, consistent with concentration in topographically heterogeneous, relatively dry landscapes. Endemic richness increased with elevation and roughness, but decreased with temperature, past‐climatic stability index, and aridity index, indicating that endemics are concentrated in high‐elevation, topographically heterogeneous, cooler, climatically stable, and relatively dry landscapes. Both components peaked in southern Anatolia (the Western and Central Taurus Mountains) and along the Anatolian Diagonal, where topographically complex, climatically stable mountain systems simultaneously function as museums of ancient lineages and cradles of new diversity, identifying these regions as priority areas for safeguarding the evolutionary potential of the Anatolian flora.

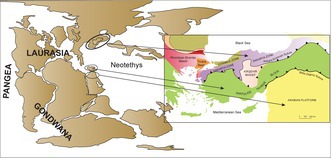

## 1 Introduction

Species richness, the number of species occurring within a defined area, is a fundamental component of biodiversity and provides the primary data for quantifying and explaining large‐scale biogeographical patterns, such as the latitudinal diversity gradient, and for testing macroecological and evolutionary theories (Whittaker 1972; Magurran 2003). Understanding the mechanisms that shape such patterns requires integrating ecological, evolutionary, and biogeographical drivers (Rosenzweig 1995; Hawkins et al. 2003; Wiens and Donoghue 2004; O'Brien 2006; Sandel et al. 2011; Stein et al. 2014; Kennedy et al. 2019). Assessing how these large‐scale drivers operate in natural systems is best achieved in regions with complex geological and climatic histories, where eco‐geographical isolation and climatic stress, such as aridity (Stebbins Jr 1952), can act as powerful drivers of diversification.

Over time, the geological evolution of a region translates into strong environmental gradients that continue to shape biodiversity patterns today. The interplay between uplift, erosion, and climatic oscillations has generated sharp variations in elevation, slope, and relief, thereby increasing topographic and habitat heterogeneity, a key determinant of species richness and endemism (Stein et al. 2014; Antonelli et al. 2018; Rahbek et al. 2019). Building on this physical template, climate influences biodiversity patterns through the interplay of energy–water dynamics, seasonal climatic variability, and ecosystem productivity (Currie 1991; Hawkins et al. 2003; O'Brien 2006; Kreft and Jetz 2007). Long‐term climatic stability has promoted in situ diversification and endemism by reducing extinction risk in refugial areas (Sandel et al. 2011; Svenning et al. 2015). Human activities further reshape biodiversity patterns through pervasive land‐use change and habitat modification (Ellis 2011; Kennedy et al. 2019). Together, these interacting environmental factors (topography, climate, stability, and human influence) explain much of the spatial variation in biodiversity.

**FIGURE 1:**
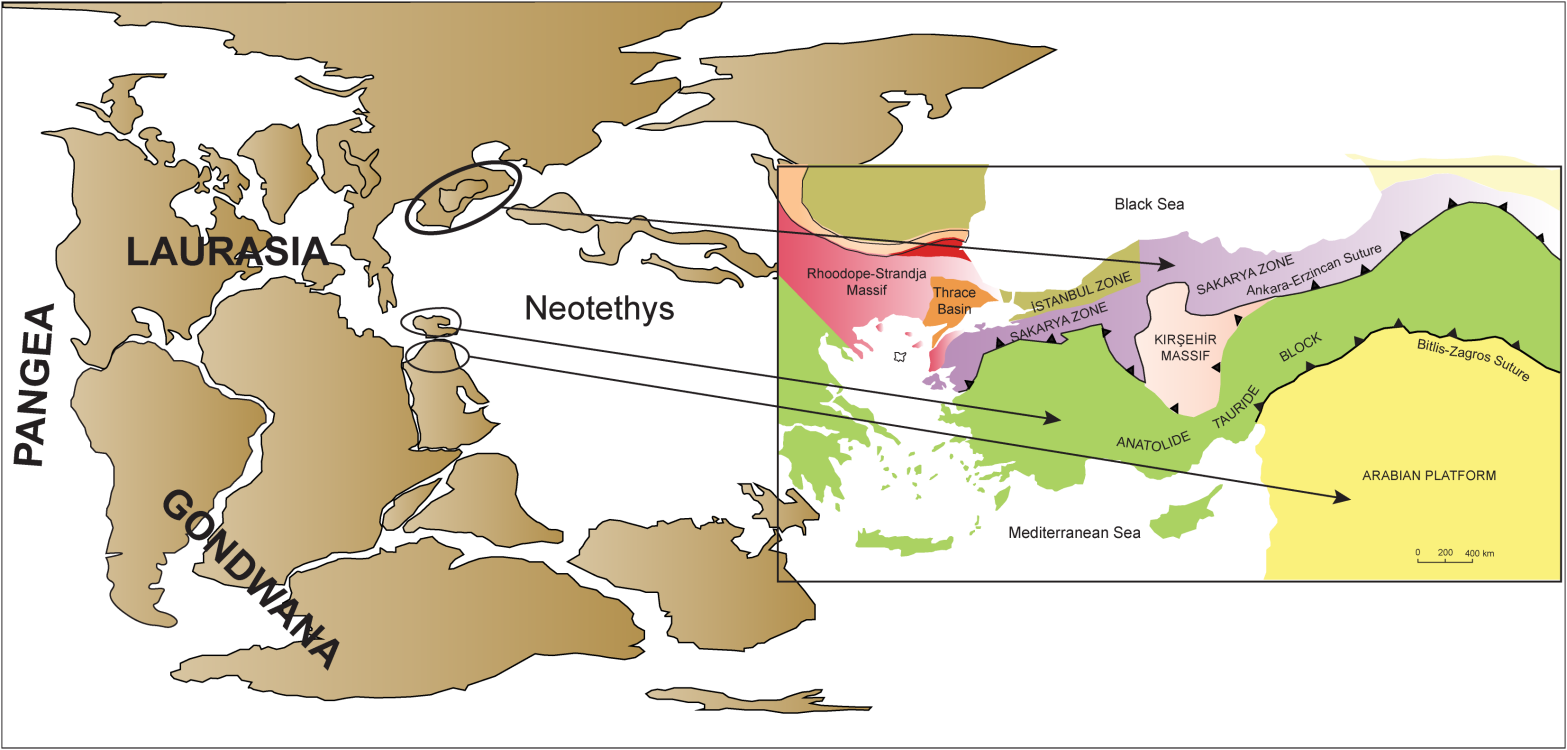
Geological evolution and tectonic framework of Anatolia. Paleogeographic reconstruction at approximately 200 Ma (main panel; modified from the GPlates portal); arrows indicate the approximate source regions of continental fragments derived from Laurasia and Gondwana that later formed Anatolia. The inset shows the present‐day distribution of major tectonic units (modified from Okay and Tüysüz 1999; Okay 2008). Anatolia emerged through the convergence of these fragments, with the Pontides (including the Sakarya and İstanbul zones) in the north and the Anatolide–Tauride Block, Kırşehir Massif, and Arabian Platform in the south. These units were amalgamated during the progressive closure of the Neotethys Ocean, now marked by major suture zones (İzmir–Ankara–Erzincan and Bitlis–Zagros sutures). This tectonic assembly underpins Anatolia's pronounced topographic complexity and substrate diversity, which provide the physical template for the region's sharp environmental gradients.

**FIGURE 2:**
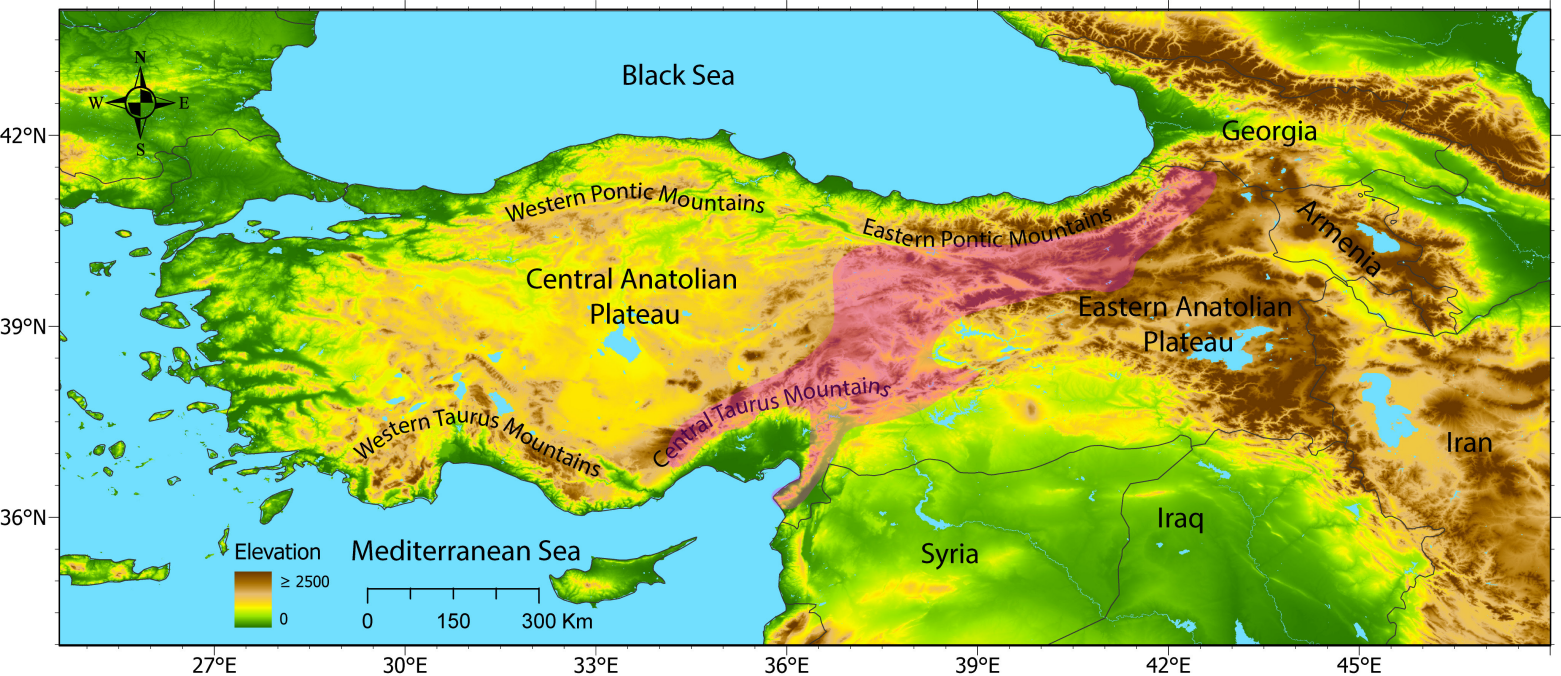
Topographic setting of Türkiye. Shaded relief shows elevation (m) from lowlands (green) to high mountains (brown), with the Anatolian Diagonal highlighted in pink. Major physiographic units, including the Western and Eastern Pontic Mountains, the Western and Central Taurus Mountains, the Central Anatolian Plateau, and the Eastern Anatolian Plateau, are indicated.

Situated at the convergence zone of major continental plates, Anatolia (the Asian part of Türkiye) offers an outstanding case for examining how geological and climatic dynamics, by generating and modulating the environmental factors outlined above, shape biodiversity patterns. The modern geological framework of Anatolia emerged through the convergence of continental fragments derived from both Laurasia (Pontides in the north) and Gondwana (Anatolide–Tauride Block, Kırşehir Massif, and Arabian Platform in the south). The collision of these fragments, following the closure of the Neotethys Ocean, placed Anatolia at the heart of the Alpine–Himalayan mountain belt, where oceanic crust was thrust onto the continents as ophiolites, mountain ranges rose, and extensive intermontane basins developed (Şengör and Yilmaz 1981; Robertson and Dixon 1984; Okay and Tüysüz 1999; Okay 2008) (Figure 1). This tectonic assembly also generated a striking substrate diversity: the juxtaposition of ophiolitic (serpentinized ultramafic) substrates, evaporitic units (e.g., gypsum‐bearing sediments), and marine carbonate rocks (limestones) produced an exceptional edaphic and geochemical mosaic across Anatolia (Dilek and Furnes 2019; Kuzucuoğlu et al. 2019; Nazik et al. 2019). Against this tectonic and edaphic backdrop, shaped by multi‐phase uplift and tectonic restructuring (McNab et al. 2018; Okay et al. 2020), Anatolia's present‐day geography is dominated by the Pontic Mountains in the north and the Taurus Mountains in the south, which enclose the elevated Central Anatolian Plateau; eastward, these systems converge into the topographically dissected, high‐elevation Eastern Anatolian Plateau. A key biogeographical feature is the Anatolian Diagonal, a mountain corridor extending from the Central Taurus Mountains and the Levant Ranges to the Eastern Pontic Mountains, widely recognized as a major barrier and filter for plant dispersal (Davis 1971; Ekim and Güner 1986; Gür 2016; Dumlupınar et al. 2023) (Figure 2). Together, the tectonic assembly, multi‐phase uplift, the resulting mountain–basin physiography, and a pronounced edaphic mosaic have shaped Anatolia into a highly elevated landscape characterized by sharp climatic and habitat gradients.

In this setting, Anatolia represents one of the most biologically diverse regions, encompassing three major phytogeographical regions: Euro‐Siberian, Irano‐Turanian, and Mediterranean (Davis 1965, 1971; Takhtajan 1986), and hosting three global biodiversity hotspots: the Mediterranean Basin, the Irano‐Anatolian, and the Caucasus (Myers et al. 2000; Mittermeier et al. 2004; Gür 2017). This phytogeographical mosaic is reflected in the flora, which comprises ~10,000 vascular plant species, of which ~3000 are endemic to Türkiye (Güner et al. 2012; Ekim 2014). Türkiye is also recognized as one of the six global priority areas among the 33 plant diversity “dark spots,” where species richness is high, but scientific knowledge remains limited (reflecting its high potential for new species descriptions and georeferenced occurrences, i.e., the Linnaean and Wallacean shortfalls) (Ondo et al. 2024). Moreover, Türkiye is identified as one of only 15 countries that collectively harbor ~50% of global plant species richness, and one of 33 countries that collectively represent ~50% of global phylogenetic diversity (Tietje et al. 2023).

Despite this global importance, Türkiye still lacks a comprehensive analysis of how species and endemic richness vary spatially across the country, and of the environmental drivers that underlie these patterns, even though a few studies have mapped endemism in particular taxa (Noroozi et al. 2019). While species richness is a fundamental component of biodiversity, endemic richness highlights areas of restricted‐range taxa and high irreplaceability, reflecting unique evolutionary history and ecological specialization (e.g., Jansson 2003; Slatyer et al. 2013; Hobohm 2014). Analyzing both metrics jointly helps distinguish drivers of broadly distributed diversity from mechanisms, such as topographic isolation and long‐term climatic stability, that promote speciation and lineage persistence (Sandel et al. 2011; Harrison and Noss 2017; Rahbek et al. 2019).

In this context, Anatolia's complex geological history and the environmental gradients it has produced make the region critically important for understanding the processes that shape biodiversity patterns at regional scales. To address this gap, we focused on the Brassicaceae, a family for which Anatolia represents a major center of diversification and endemism (Franzke et al. 2011). Türkiye hosts 602 Brassicaceae species, including 228 endemics (~38%), placing the family among the country's leading plant lineages both in total species richness and in the proportion of endemic species (ranked third; Güner et al. 2012; Ekim 2014). Spanning alpine, steppe, Mediterranean, and serpentine habitats (Davis 1965; Güner et al. 2012), the family's exceptional ecological breadth and evolutionary plasticity—driven by chromosomal rearrangements (Lysak et al. 2006; Guo et al. 2021; Jiang et al. 2025), repeated radiations, and adaptive responses to environmental change (Franzke et al. 2011; Huang et al. 2020; Walden et al. 2020)–make it particularly suitable for testing how environmental gradients shape species and endemic richness patterns.

To this end, we compiled a comprehensive occurrence dataset for Brassicaceae across Türkiye, comprising > 15,000 unique, georeferenced records after quality control. We then selected a set of environmental predictors with the potential to influence biodiversity patterns and implemented a spatially explicit analytical framework to evaluate their effects on species and endemic richness, thereby providing a quantitative test of the long‐standing idea that eco‐geographical isolation and aridity have played a central role in the diversification of the Anatolian flora (Davis 1971). Within this framework, we addressed three key questions: (i) How do species and endemic richness vary spatially? (ii) To what extent are these patterns explained by environmental versus spatial processes? and (iii) Do the environmental drivers of species richness differ from those of endemic richness? We hypothesized that environmental processes would emerge as the dominant correlates of both species and endemic richness, whereas spatial processes alone would play only a minor role.

## 2 Methods

### 2.1 Data and Spatial Framework

We compiled a comprehensive occurrence dataset for Brassicaceae in Türkiye, a recognized center of diversification and endemism for the family (Franzke et al. 2011). First, we prepared an updated checklist of all taxa (species, subspecies, and varieties) native to Türkiye (see Data Availability Statement). As a baseline, we followed the “Türkiye Bitkileri Listesi” (Güner et al. 2012). Tribal and generic limits were initially taken from Brassibase (Kiefer et al. 2014). Because Brassibase has not been consistently updated during the study period, we revised the checklist using Plants of the World Online (POWO 2025) and other recent taxonomic treatments published in the literature (Özhatay et al. 2013, 2016, 2017, 2019; Španiel et al. 2015; Özgişi et al. 2018; Özçandir et al. 2019; Özgişi 2020; German and Özüdoğru 2020; Ertuğrul et al. 2021; Hamzaoglu and Koc 2022; Özdöl et al. 2022; Öztürk 2022; Tunçkol et al. 2022).

For each accepted taxon with available occurrence data, we assembled records from multiple sources. We extracted all Brassicaceae records from *Flora of Turkey and the East Aegean Islands* (Davis 1965; Davis et al. 1988; Güner et al. 2000), yielding 2532 records. We then carried out extensive herbarium work to expand and update these data. Physical herbarium visits were conducted at major Turkish collections (AKDU, ANK, EGE, GAZI, HUB, ISTE, and NGBB), yielding 11,432 records (Table S1). For each Brassicaceae specimen, we photographed the label and recorded the taxon name, locality description, geographic coordinates (if provided), elevation, collection date, collector, and determiner. In addition, we incorporated digitized Brassicaceae specimens from major international herbaria with extensive Turkish holdings (B, E, and W), adding 5257 records. In total, herbarium work contributed 16,689 records, and we obtained 19,221 records before filtering (Table S1). We then performed quality control: records with unclear or unusable locality information were removed, and duplicate records referring to the same physical specimen cited across sources were identified and removed. After cleaning, 15,547 unique records remained (Figure S1, see Data Availability Statement). Because the checklist summarizes the overall Brassicaceae flora native to Türkiye, whereas the spatial models are based on the subset of taxa for which specimen‐supported georeferenced records were available after quality control, some checklist taxa lack usable records at our spatial resolution, an expected feature of herbarium‐based biogeographical analyses.

For each specimen, if geographic coordinates were present on the label, we used them directly; otherwise, we manually georeferenced the locality description, reconciling historical or outdated toponyms with published gazetteers and other geographic references (Kandemir 2014) and identifying the most precise locality consistent with elevation and habitat information. This georeferencing protocol is scaled to the study's spatial resolution and provides an appropriate trade‐off between positional uncertainty in historical herbarium labels and spatial precision.

**FIGURE 3:**
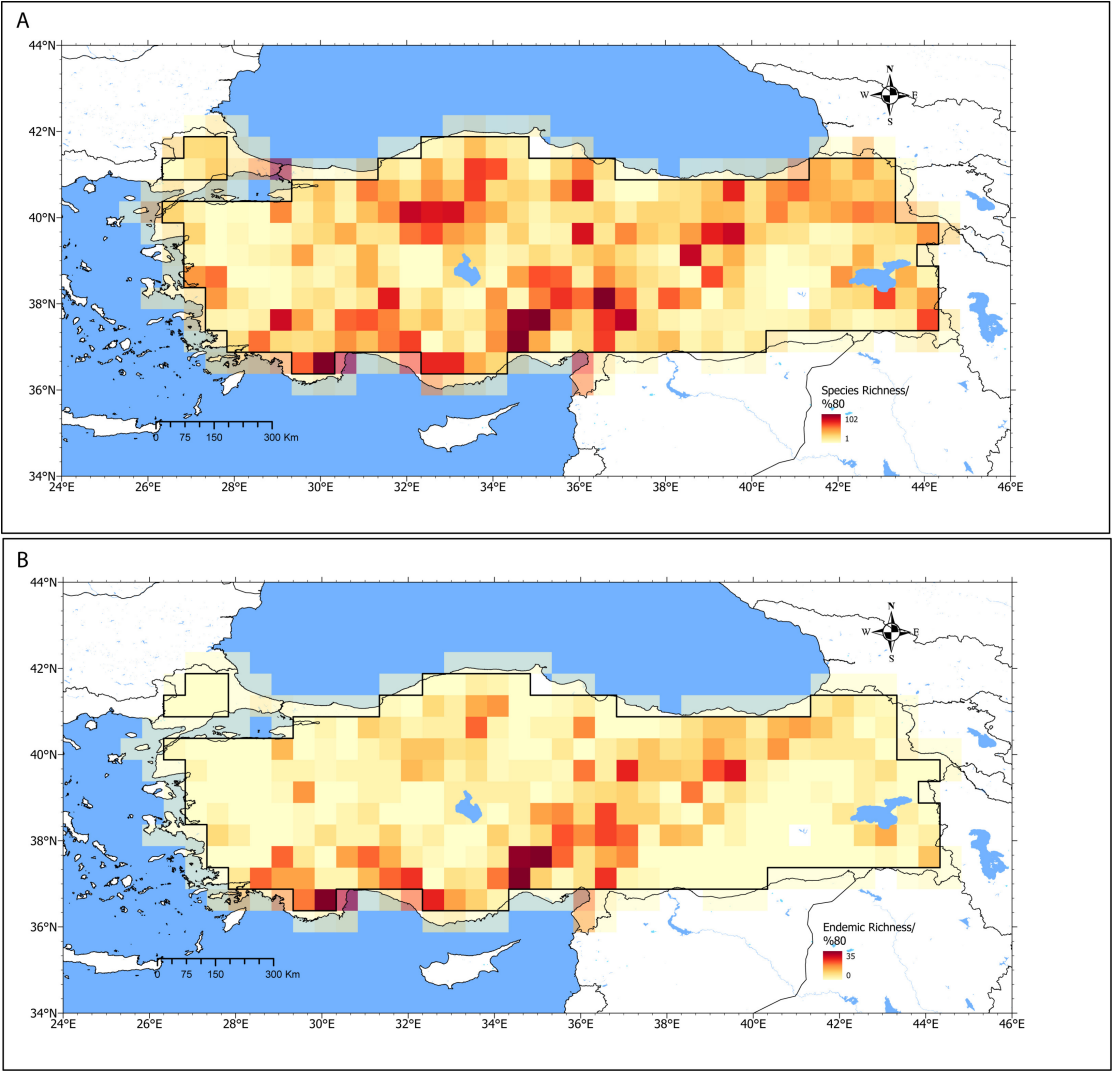
Spatial patterns of Brassicaceae richness in Türkiye. (A) Species richness and (B) endemic richness, expressed as the number of taxa per 0.5° × 0.5° grid cell. Warm colors indicate higher richness. Given Türkiye's intricate coastline and large inland lakes (Lake Tuz and Lake Van), we retained only grid cells with ≥ 80% terrestrial area; these cells are delineated by the solid black outline and yielded a working grid of 286 cells used in all subsequent analyses.

To analyze geographic patterns, Türkiye was divided into a grid of 0.5° × 0.5° cells (Figure 3, Figure S1). The grid resolution was chosen to balance two constraints: (i) biological/biogeographical relevance at the national scale and (ii) positional uncertainty in historical herbarium labels, which often lack precise geographic coordinates. Given Türkiye's intricate coastline and borders, and the presence of two large lakes (Lake Tuz and Lake Van), we excluded grid cells with < 80% terrestrial area within Türkiye (i.e., cells dominated by water or by land outside Türkiye's borders); this reduced the working grid from 384 to 286 cells (Figure 3). Sensitivity checks with alternative thresholds yielded similar results, and we adopted the 80% criterion to maximize cell coverage while minimizing mixed land–water and cross–border cells. Although the grid‐based analyses follow Türkiye's national boundaries, the European part of Türkiye (Thrace) is represented by only a few grid cells; accordingly, our biogeographical inferences primarily reflect Anatolia.

Georeferencing, gridding, and spatial preprocessing were performed in Google Earth Pro 7.3.6 and ArcGIS Pro 3.0.0 (Esri), whereas data processing and table management were carried out in R v4.3.3 (R Core Team 2024) using the readxl v1.4.4 (Wickham and Bryan 2025), dplyr v1.1.4 (Wickham et al. 2023), and tidyr 1.3.1 (Wickham et al. 2024) packages.

### 2.2 Response Variables

We calculated two response variables for each 0.5° × 0.5° grid cell, (i) species richness (number of species) and (ii) endemic richness (number of endemic species), in ArcGIS Pro 3.0.0 using SDMtoolbox v.9.1 (Brown 2014) (Figure 3, see Data Availability Statement). Because species richness contained no zeros, we applied log10(species richness); endemic richness included zeros, so we used log10(1 + endemic richness). These transformations mitigated right‐skewness (and zero inflation for endemic richness) and stabilized variance. Standard diagnostics (residual maps, histograms, Q–Q plots, and residuals‐versus‐fitted plots), Moran's I on residuals, and Breusch–Pagan/Koenker–Bassett tests supported the transformations and the statistical modeling choices, specifically the use of Gaussian spatial regression models (Ives 2015).

### 2.3 Environmental Predictors

To capture a broad range of contemporary, historical, and anthropogenic factors potentially influencing species and endemic richness, we considered the following environmental predictors (see Data Availability Statement): mean annual temperature (BIO1), temperature seasonality (BIO4), annual precipitation (BIO12), precipitation seasonality (BIO15), aridity index (AI), a general past‐climatic stability index (CSI), past‐climatic stability indices for mean annual temperature and annual precipitation (CSI‐BIO1 and CSI‐BIO12, respectively), net primary production (NPP), human modification (HM), elevation, and topographic roughness. Higher values of the climatic stability indices (CSI, CSI‐BIO1, CSI‐BIO12) indicate lower long‐term climatic stability (i.e., greater past climatic variability). Similarly, higher values of the aridity index (AI) indicate wetter conditions (i.e., increasing water availability). Climate layers (BIO1, BIO4, BIO12, BIO15, AI) were obtained from Karger et al. (2017); past‐climatic stability layers (CSI, CSI‐BIO1, CSI‐BIO12) from Herrando‐Moraira et al. (2022); net primary production (NPP) from MODIS (MOD17A3H v6); human modification (HM) from Theobald et al. (2025); and elevation and topographic roughness from Amatulli et al. (2018) (Figure S2; Table S2). All layers were resampled from their native resolutions to the study's 0.5° × 0.5° grid and then standardized (z‐scores), enabling coefficients to be directly comparable in terms of effect size.

We assessed collinearity among environmental predictors using variance inflation factors (VIF) and constrained model specification to VIF ≤ 5 (Zuur et al. 2010). To limit multicollinearity, we did not include BIO1 with elevation or BIO12 with AI in the same model. Accordingly, we defined four alternative predictor sets: (i) BIO1 + AI, (ii) BIO1 + BIO12, (iii) elevation + AI, and (iv) elevation + BIO12, together with a common set of covariates (BIO4, BIO15, CSI, CSI‐BIO1, CSI‐BIO12, HM, NPP, roughness). We then fit the statistical models for each candidate set.

### 2.4 Spatial Weights Matrix

The study grid is not fully contiguous, so we defined spatial neighbors with a centroid‐based k‐nearest neighbors (kNN) scheme and symmetrized (bidirectional) kNN to obtain a connected, undirected network. We adopted *k* = 6 as the baseline to avoid isolates without introducing unrealistically long connections. Sensitivity checks at *k* = 4 and *k* = 8 yielded qualitatively similar results, supporting the robustness of the spatial weighting scheme. Neighbor weights were row‐standardized (W‐style), and edge cells were handled consistently. All neighbor construction and spatial weighting were performed in R v4.3.3 using the spdep package v1.4‐1 (Bivand 2022).

### 2.5 Model Fitting

For each candidate set, we first fit four model types: one non‐spatial baseline (ordinary least squares, OLS) and three spatial regression models (spatial autoregressive, SAR; spatial error SEM; and spatial autoregressive combined, SAC) (Dormann et al. 2007; Kissling and Carl 2008) to account for spatial autocorrelation in environmental conditions and biological responses across neighboring grid cells (Legendre 1993). Model choice was based on Akaike Information Criterion (AIC, treating ΔAIC < 2 as equally supported) while prioritizing parsimony and diagnostics. Adequacy checks included standard diagnostics (residual maps, histograms, Q–Q plots, residuals‐versus‐fitted plots), Monte Carlo simulations of Moran's I on residuals (*n* = 2000), and Breusch–Pagan/Koenker–Bassett tests for heteroskedasticity. In addition, we computed Rao's score (Lagrange Multiplier) tests from the OLS residuals (LM‐lag/LM‐error and their robust variants) to diagnose and corroborate the form of spatial dependence underlying the AIC‐guided choice (e.g., a significant robust lag with a non‐significant robust error supports SAR). For the SAR models, we further decomposed the estimated effects into direct, indirect (spatial spillover), and total impacts. The significance of each component was assessed with Monte Carlo simulations (*n* = 2000). All modeling was conducted in R v4.3.3 using the spdep and spatialreg v1.4‐2 (Bivand et al. 2021) packages. To assess sensitivity to the neighbor definition, we repeated the full procedure with bidirectional kNN graphs at *k* = 4 and *k* = 8; signs and magnitudes of key effects were qualitatively unchanged, supporting the robustness of the spatial weighting scheme.

We quantified the unique and shared contributions of environment and space to species and endemic richness for the best‐supported and parsimonious models using variation partitioning based on partial redundancy analysis (RDA) (Peres‐Neto et al. 2006). The environmental matrix E comprised environmental predictors used in model fitting. Spatial structure S was summarized by the first two principal components of centered‐and‐scaled cell‐centroid coordinates, capturing broad‐scale spatial trends. We partitioned variance into fractions explained uniquely by E (E|S), uniquely by S (S|E), jointly by E∩S, and unexplained. Significance of E|S and S|E was assessed with permutation tests (*n* = 2000) on partial RDA models under the reduced model. Because richness is a univariate response, RDA is equivalent to partial multiple regression; accordingly, we report adjusted *R* ^2^ for explained fractions. All analyses were performed in R v4.3.3 using the vegan v2.7‐2 package (Oksanen et al. 2025).

We applied geographically weighted regression (GWR) (Brunsdon et al. 1996) to explore spatial variation in the relationships between species and endemic richness and environmental predictors for the best‐supported and parsimonious models, with the final GWR models selected based on the corrected Akaike Information Criterion (AICc, treating ΔAICc < 2 as equally supported). Geographic coordinates were projected to ETRS89/LAEA Europe (EPSG:3035) to ensure metric distances. An adaptive bisquare kernel was used, and the optimal bandwidth was selected by minimizing AICc. To ensure the stability of local parameter estimates and rule out local multicollinearity, we calculated local condition numbers (CN); CN values remained within acceptable limits across the study area, confirming that the local parameter estimates were not degraded by multicollinearity. The statistical significance of spatial variability in the coefficients was assessed using Monte Carlo simulations, and local *R* ^2^ values were mapped to show spatial variation in model explanatory power. GWR was implemented in R v4.3.3 using the Gwmodel v2.4‐1 package (Gollini et al. 2015).

*p* values were adjusted using the false‐discovery‐rate (FDR) correction across all environmental predictors to account for multiple comparisons.

### 2.6 Model Visualization

We visualized relationships between species and endemic richness and environmental predictors using partial plots based on the OLS model, illustrating the independent contribution of each predictor while controlling for all others. These plots aid interpretation of individual effects under the multiple regression framework and provide a baseline against which to compare results from the spatial models. Additionally, bivariate scatterplots between untransformed variables were included for exploratory purposes, showing overall distributional patterns and data structure prior to transformation and modeling. All plots were produced in R v4.3.3 using the car v3.1‐3 (Fox and Weisberg 2019) and ggplot2 v4.0.0 (Wickham 2011) packages.

## 3 Results

### 3.1 Taxonomic Overview

An updated checklist of the Brassicaceae taxa naturally occurring in Türkiye was compiled. According to this checklist, the Brassicaceae flora of Türkiye is represented by 28 tribes and 96 genera (including one genus not assigned to any tribe), comprising 663 species, 79 subspecies, and 16 varieties, for a total of 758 taxa. Of these, 273 species are endemic to Türkiye, corresponding to an endemic‐species proportion of ~41.2% (see Data Accessibility Statement).

### 3.2 Species Richness

Species richness per 0.5° × 0.5° grid cell ranged from 1 to 102 species (mean = 25.89, SD = 20.32; median = 21, Q1–Q3 = 9–38, IQR = 29; *n* = 286). Species richness was highest in southern Anatolia, particularly along the Western and Central Taurus Mountains, extending through the Anatolian Diagonal, and in the northern parts of the Central Anatolian Plateau (Figure 3).

Model evaluation showed that all four candidate sets produced significant models, but model choice favored the spatial models relative to the non‐spatial baseline. Model fit likewise improved, with higher explanatory power in the spatial models. AIC values decreased markedly from the ordinary least squares (OLS) model to the spatial models. According to the ΔAIC < 2 criterion, the mean annual temperature (BIO1) + annual precipitation (BIO12) (spatial autoregressive, SAR and spatial autoregressive combined, SAC) and elevation + BIO12 (SAR) models were statistically indistinguishable and therefore considered equally supported. Both SAR models were retained as the most parsimonious ones, because the SAC alternative, although nearly identical in coefficients and spatial‐lag parameter (ρ), required an additional parameter (λ, the spatial‐error parameter), which was non‐significant, further supporting our model choice. The spatial‐lag parameter was highly significant in both SAR models, confirming a strong spatial component in species richness (Table 1). Standard diagnostics (residual maps, histograms, Q–Q plots, and residuals‐versus‐fitted plots) showed no systematic deviation from normality or homoscedasticity, and Breusch–Pagan tests were consistent with homoscedasticity (for both SAR models, *χ* ^2^ ≥ 14.76, df = 10, *p* ≥ 0.077). Rao's score (Lagrange Multiplier) diagnostics on the OLS residuals (for both SAR models, robust LM‐lag ≥ 12.99, df = 1, *p* < 0.001; robust LM‐error ≥ 1.94, df = 1, *p* ≥ 0.122) supported a spatial‐lag specification and indicated no additional spatial‐error component, justifying our focus on SAR models. Monte Carlo distributions of Moran's I likewise confirmed that spatial autocorrelation was effectively removed. Collectively, these results demonstrated that spatial dependence was properly modeled, and that the SAR models provided the most parsimonious and statistically robust explanation of species richness patterns (Table 1).

**TABLE 1:** Summary of non‐spatial (OLS) and spatial regression models (SAR, SEM and SAC) for four alternative predictor sets: (i) BIO1 + AI, (ii) BIO1 + BIO12, (iii) elevation + AI, and (iv) elevation + BIO12, each fitted together with a common set of covariates (BIO4, BIO15, CSI, CSI‐BIO1, CSI‐BIO12, HM, NPP, and roughness) for Brassicaceae species and endemic richness in Türkiye. AIC, ΔAIC, adjusted *R* ^2^, and Moran's I are given as ranges for the corresponding non‐spatial OLS models fitted with the same predictor sets as the supported spatial models. Only the best‐supported spatial models (ΔAIC < 2) are shown; SEM models and other higher‐AIC alternatives are omitted.

Response	Model (predictors)	Spatial parameter (*ρ*/λ)	AIC	ΔAIC	Adjusted‐*R* ^2^/pseudo‐*R* ^2^	Moran's I (residuals)
Species richness	OLS (Baseline)	—	293.21–293.72	31.70–32.21	0.20	0.16***
	SAR (BIO1 + BIO12)	*ρ* = 0.50***	261.51	0	0.34	0 n.s.
	SAR (Elevation + BIO12)	*ρ* = 0.50***	262.88	1.37	0.34	0 n.s.
	SAC (BIO1 + BIO12)	*ρ* = 0.55***, λ = −0.09 n.s.	263.38	1.87	0.35	0 n.s.
Endemic richness	OLS (Baseline)	—	163.74–171.06	26.23–33.55	0.40–0.41	0.12–0.14***
	SAR (Elevation + AI)	*ρ* = 0.43***	137.51	0	0.50	−0.01 n.s.
	SAR (BIO1 + AI)	*ρ* = 0.47***	138.06	0.55	0.50	−0.02 n.s.
	SAC (BIO1 + AI)	*ρ* = 0.56***, λ = −0.23 n.s.	138.65	1.14	0.52	0 n.s.
	SAC (Elevation + AI)	*ρ* = 0.49***, λ = −0.14 n.s.	138.97	1.46	0.51	0 n.s.

*Note:* BIO1, Mean annual temperature. BIO4, Temperature seasonality. BIO12, Annual precipitation. BIO15, Precipitation seasonality. CSI, General past‐climatic stability index. CSI‐BIO1, Past‐climatic stability index for mean annual temperature. CSI‐BIO12, Past‐climatic stability index for annual precipitation. ρ (rho), Spatial lag parameter in SAR/SAC models. λ (lambda), Spatial error parameter in SAC models. ΔAIC, Difference in AIC relative to the best‐supported model. Adjusted‐*R* ^2^, Adjusted coefficient of determination in OLS models. Pseudo‐*R* ^2^, Pseudo R‐squared in SAR/SAC models.

Abbreviations: AI, aridity index; AIC, akaike's information criterion; HM, human modification; n.s., not significant; NPP, net primary production; OLS, ordinary least squares; SAC, spatial autoregressive combined; SAR, spatial autoregressive; SEM, spatial error.

*** *p* < 0.001.

**FIGURE 4:**
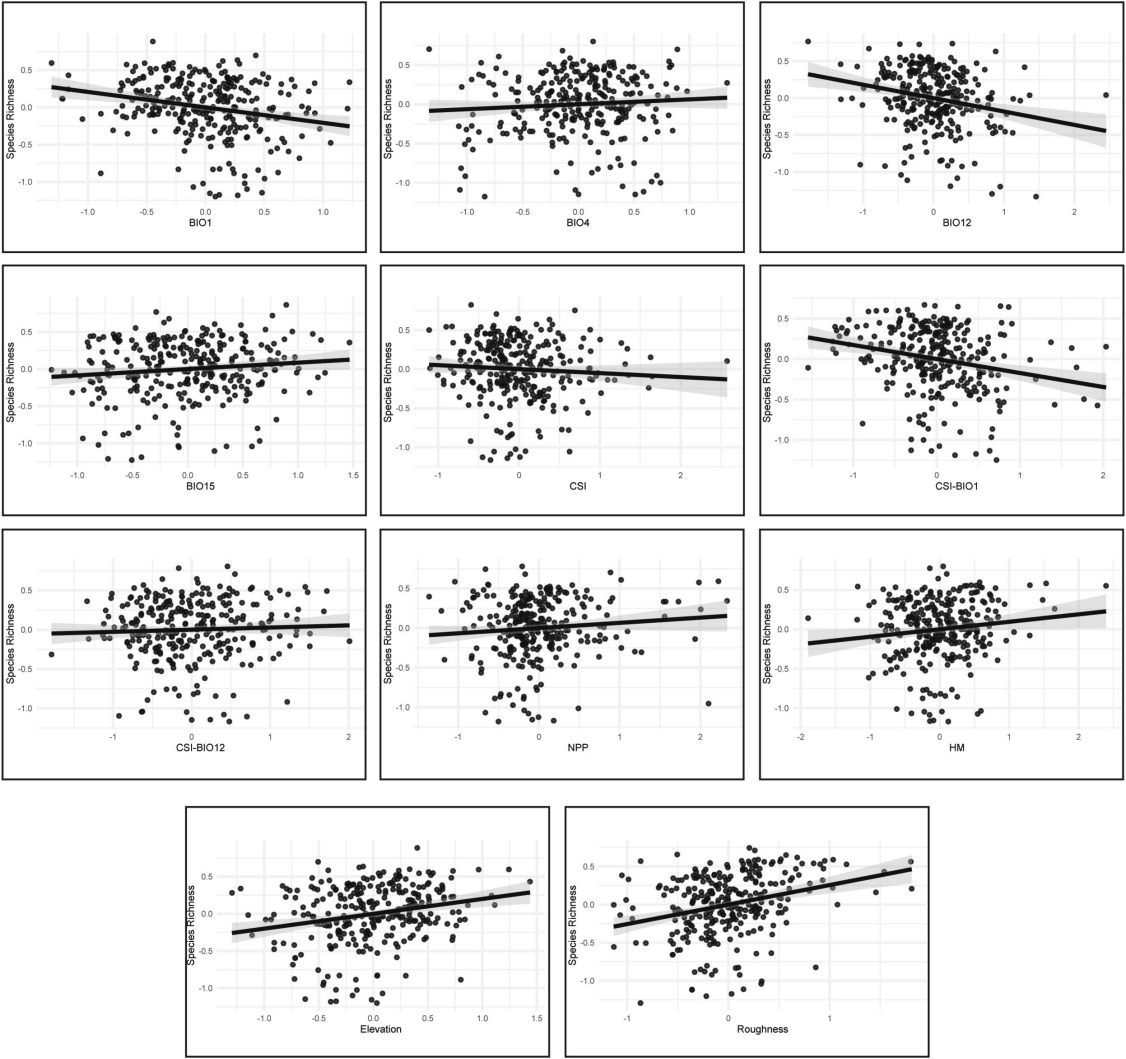
Partial regression plots for Brassicaceae species richness in Türkiye. Each panel shows the partial relationship between species richness and a single environmental predictor (BIO1, BIO4, BIO12, BIO15, CSI, CSI‐BIO1, CSI‐BIO12, NPP, HM, elevation, and roughness), after controlling for all remaining predictors. The set of predictors corresponds primarily to those included in the best‐supported SAR model for species richness (BIO1 + BIO12), while the plot for elevation is taken from the alternative best‐supported SAR model (see Table 1). Axes show standardized residuals of species richness (y‐axis) and the focal predictor (x‐axis). The solid line is the fitted linear relationship, and the shaded envelope denotes the 95% confidence interval. BIO1, Mean annual temperature. BIO4, Temperature seasonality. BIO12, Annual precipitation. BIO15, Precipitation seasonality. CSI, General past‐climatic stability index. CSI‐BIO1, Past‐climatic stability index for mean annual temperature. CSI‐BIO12, Past‐climatic stability index for annual precipitation. NPP, Net primary production. HM, Human Modification.

The two best‐supported SAR models identified two environmental predictors with robust and consistent total effects: topographic roughness and BIO12. Direct (local) and indirect (neighbor‐mediated) components were directionally consistent, confirming coherent spatial spillover patterns. Roughness had a strong positive total effect, indicating that species richness increases in topographically more heterogeneous areas. Conversely, BIO12 had a strong negative total effect, indicating that species richness decreases in wetter areas (Table 2, Figure 4, Figure S3).

**TABLE 2:** Direct, indirect, and total SAR impacts of environmental predictors on Brassicaceae species and endemic richness in Türkiye. SAR impacts are primarily reported from the best‐supported SAR model for each response (species richness: BIO1 + BIO12; endemic richness: Elevation + AI). For species richness, the impact estimates for elevation, and for endemic richness, those for BIO1 are taken from the alternative best‐supported SAR model fitted for that response (see Table 1). Dashes (—) indicate that the predictor was not included in any of the best‐supported SAR models for that response variable.

Predictors	Species richness			Endemic richness		
	Direct	Indirect	Total	Direct	Indirect	Total
BIO1	−0.126	−0.116	−0.241 n.s.	−0.247	−0.198	−0.445***
BIO4	0.018	0.016	0.034 n.s.	0.003	0.002	0.006 n.s.
BIO12	−0.161	−0.149	−0.310**	—	—	—
BIO15	0.083	0.076	0.159 n.s.	0.025	0.018	0.043 n.s.
AI	—	—	—	−0.096	−0.068	−0.164*
CSI	−0.031	−0.028	−0.059 n.s.	−0.083	−0.059	−0.142*
CSI—BIO1	−0.089	−0.082	−0.172 n.s.	−0.119	−0.084	−0.204***
CSI—BIO12	0.028	0.026	0.054 n.s.	−0.034	−0.024	−0.058 n.s.
NPP	0.066	0.061	0.128 n.s.	0.045	0.031	0.076 n.s.
HM	0.063	0.058	0.121 n.s.	0.034	0.024	0.058 n.s.
Elevation	0.109	0.100	0.209 n.s.	0.245	0.173	0.417***
Roughness	0.186	0.171	0.357**	0.179	0.126	0.305***

*Note:* The only total SAR impacts, not direct or indirect components, were tested for statistical significance. BIO1, Mean annual temperature. BIO4, Temperature seasonality. BIO12, Annual precipitation. BIO15, Precipitation seasonality. CSI, General past‐climatic stability index. CSI‐BIO1, Past‐climatic stability index for mean annual temperature. CSI‐BIO12, Past‐climatic stability index for annual precipitation.

Abbreviations: AI, aridity index; HM, human modification; n.s., not significant; NPP, net primary production; SAR, spatial autoregressive.

* *p* < 0.05.

** *p* < 0.01.

*** *p* < 0.001.

Variation partitioning revealed that environmental predictors explained a substantial portion of the variation in species richness after accounting for spatial structure. The environment‐only fraction (E|S) accounted for approximately 20% of the adjusted *R* ^2^ and was significant (for both SAR models, *F* ≥ 7.92, df = 10, *p* < 0.001), whereas the spatial‐only fraction (S|E) was negligible (0%) and non‐significant (*F* ≥ 0.44, df = 2, *p* ≥ 0.212). These results indicated that environmental gradients, rather than purely spatial structure, primarily govern species richness patterns (Table 3).

**TABLE 3:** Summary of variation partitioning for Brassicaceae species and endemic richness into environmental and spatial components for the two best‐supported SAR models (see Table 1). Values are adjusted *R* ^2^ fractions expressed as percentages of the total variation for the environment‐only [a], space‐only [b], shared [c], and unexplained [d] components. Note that all models include a common set of covariates (BIO4, BIO15, CSI, CSI‐BIO1, CSI‐BIO12, HM, NPP, and roughness).

Response	Model (predictors)	Environment [a] (%)	Space [b] (%)	Shared effect [c] (%)	Unexplained [d] (%)
Species richness	BIO1 + BIO12	20.41	0.32	−0.20	79.48
	Elevation + BIO12	19.62	−0.33	0.44	80.26
Endemic richness	Elevation, AI	40.06	−0.06	1.24	58.76
	BIO1, AI	41.36	2.76	−1.58	57.46

*Note:* [a] Environment | Space: unique environmental fraction after accounting for spatial effects. [b] Space | Environment: unique spatial fraction after accounting for environmental effects. [c] Shared: variation jointly explained by environment and space. [d] Unexplained: residual variation (1 – [a] – [b] – [c]). Small negative percentages for [b] and [c] reflect adjusted *R* ^2^ values slightly below zero and indicate fractions effectively equal to zero. BIO1, Mean annual temperature. BIO4, Temperature seasonality. BIO12, Annual precipitation. BIO15, Precipitation seasonality. CSI, General past‐climatic stability index. CSI‐BIO1, Past‐climatic stability index for mean annual temperature. CSI‐BIO12, Past‐climatic stability index for annual precipitation.

Abbreviations: AI, aridity index; HM, human modification; NPP, net primary production.

**FIGURE 5:**
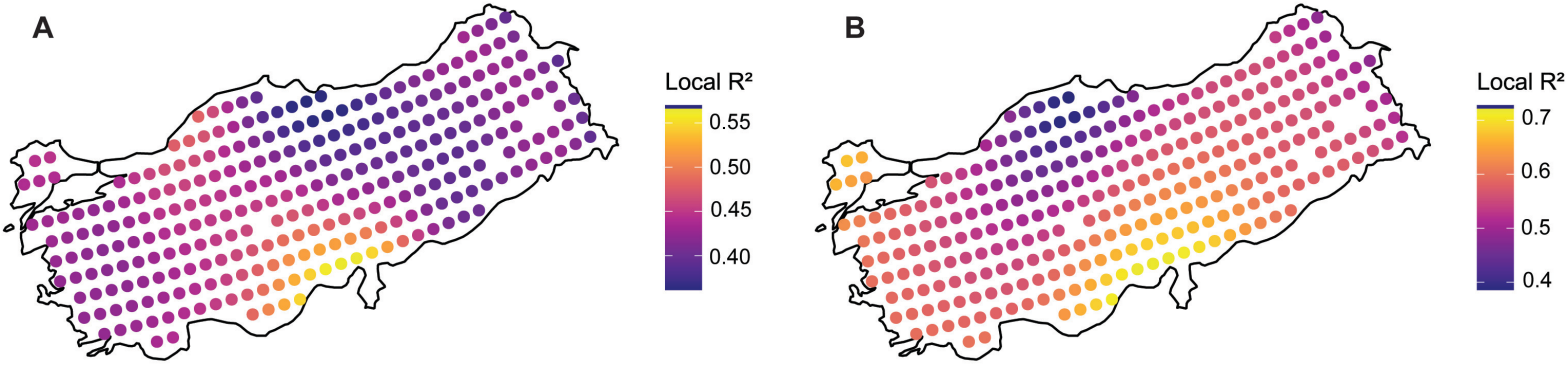
Local explanatory power (*R* ^2^) of environmental predictors for Brassicaceae richness in Türkiye, estimated using geographically weighted regression (GWR). Panels show (A) species richness and (B) endemic richness. Each circle represents a 0.5° × 0.5° grid cell. Warmer colors indicate higher local explanatory power for richness.

Geographically weighted regression (GWR) based on the BIO1 + BIO12 model, the only best‐supported model according to the ΔAICc < 2 criterion, revealed substantial spatial heterogeneity in the relationships between species richness and environmental predictors. Local model performance varied widely across Türkiye, with a mean local *R* ^2^ of 44%, considerably higher than the global pseudo‐*R* ^2^ (34%), indicating marked spatial variation in explanatory power. The highest local *R* ^2^ values occurred in southern Anatolia, particularly along the Central Taurus Mountains, the most species‐rich region, where model fit often exceeded 55% (Figure 5).

### 3.3 Endemic Richness

Endemic richness per 0.5° × 0.5° grid cell ranged from 0 to 35 species (mean = 4.95, SD = 5.72; median = 3, Q1–Q3 = 1–7, IQR = 6; *n* = 286). Endemic richness was also highest in southern Anatolia, particularly along the Western and Central Taurus Mountains, extending through the Anatolian Diagonal (Figure 3).

Model evaluation similarly showed that all four candidate sets produced significant models, but model choice again favored the spatial models relative to the non‐spatial baseline. Model fit likewise improved, with higher explanatory power in the spatial models. AIC values decreased markedly from the OLS model to the spatial models. According to the ΔAIC < 2 criterion, the elevation + aridity index (AI) (SAR and SAC) and BIO1 + AI (SAR and SAC) models were statistically indistinguishable and therefore considered equally supported. Both SAR models were retained as the most parsimonious ones, because the SAC alternatives, although nearly identical in coefficients and spatial‐lag parameter (ρ), included a non‐significant spatial‐error parameter (λ). The spatial‐lag parameter was highly significant in both SAR models, confirming a strong spatial component in endemic richness (Table 1). Standard diagnostics again showed no systematic deviation from normality or homoscedasticity, and Breusch–Pagan tests were consistent with homoscedasticity (for both SAR models, *χ* ^2^ ≥ 9.78, df = 10, *p* ≥ 0.453). Rao's score (Lagrange Multiplier) diagnostics on the OLS residuals (for both SAR models, robust LM‐lag ≥ 16.98, df = 1, *p* < 0.001; robust LM‐error ≥ 1.17, df = 1, *p* ≥ 0.129) supported a spatial‐lag specification and indicated no additional spatial‐error component, justifying our focus on SAR models. Monte Carlo distributions of Moran's I likewise confirmed that spatial autocorrelation was effectively removed. Together, these results demonstrated that spatial dependence was properly modeled, and that the SAR models provided the most parsimonious and statistically robust explanation of endemic richness patterns (Table 1).

**FIGURE 6:**
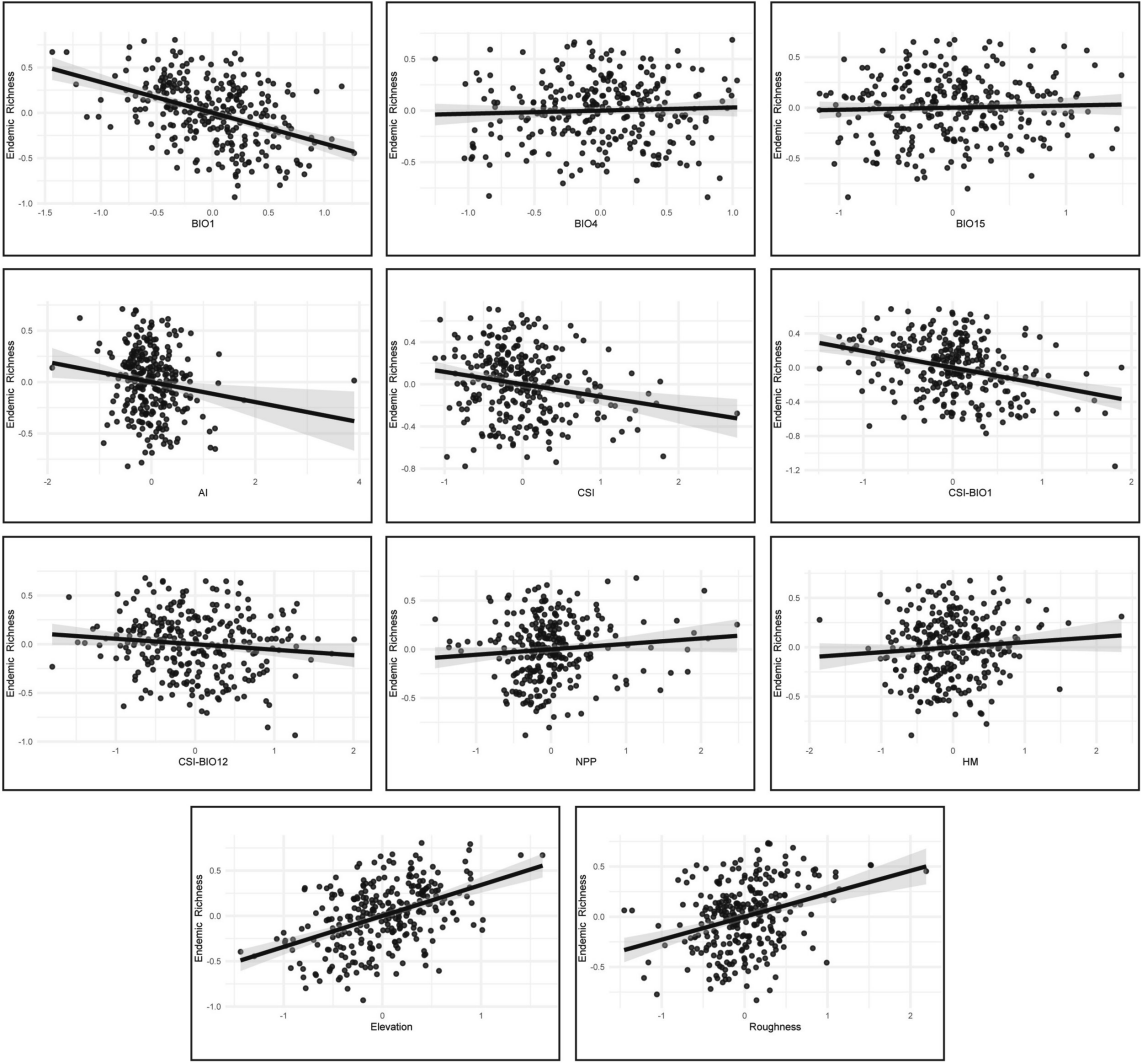
Partial regression plots for Brassicaceae endemic richness in Türkiye. Each panel shows the partial relationship between endemic richness and a single environmental predictor (BIO1, BIO4, BIO15, AI, CSI, CSI‐BIO1, CSI‐BIO12, NPP, HM, elevation, and roughness), after controlling for all remaining predictors. The set of predictors corresponds primarily to those included in the best‐supported SAR model for endemic richness (elevation + AI), while the plot for BIO1 is taken from the alternative best‐supported SAR model (see Table 1). Axes show standardized residuals of endemic richness (y‐axis) and the focal predictor (x‐axis). The solid line is the fitted linear relationship and the shaded envelope denotes the 95% confidence interval. BIO1, Mean annual temperature. BIO4, Temperature seasonality. BIO15, Precipitation seasonality. AI, Aridity index. CSI, General past‐climatic stability index. CSI‐BIO1, Past‐climatic stability index for mean annual temperature. CSI‐BIO12, Past‐climatic stability index for annual precipitation. NPP, Net primary production. HM, Human Modification.

The two best‐supported SAR models identified five environmental predictors with robust and consistent total effects: BIO1, elevation, roughness, past‐climatic stability index (general past‐climatic stability index, CSI and past‐climatic stability index for mean annual temperature, CSI‐BIO1), and AI. Direct and indirect components were directionally consistent, confirming coherent spatial spillover patterns. Elevation and roughness had strong positive total effects, indicating that endemic richness increases in high‐elevation, topographically more heterogeneous areas. Conversely, BIO1, CSI, CSI‐BIO1, and AI had strong negative total effects, indicating that endemic richness decreases in warmer, climatically unstable, and wetter areas (Table 2, Figure 6, Figure S4).

Variation partitioning revealed that environmental predictors explained a substantial portion of the variation in endemic richness after accounting for spatial structure. The environment‐only fraction (E|S) accounted for approximately 40% of the adjusted *R* ^2^ and was significant (for both SAR models, *F* ≥ 20.29, df = 10, *p* < 0.001), whereas the spatial‐only fraction (S|E) was negligible, accounting for only 2.8% of the adjusted *R* ^2^ in the BIO1 + AI model (*F* = 7.60, df = 2, *p* < 0.001) and 0% in the elevation + AI model (*F* = 0.86, df = 2, *p* = 0.438). A weak, but significant spatial component remained in the BIO1 + AI model, likely reflecting residual large‐scale geographic gradients not fully captured by the temperature‐based model, whereas the spatial component was entirely absorbed by the elevation‐based model. These results again indicated that environmental gradients, rather than purely spatial structure, primarily govern endemic richness patterns (Table 3).

GWR based on the BIO1 + AI model, the only best‐supported model according to the ΔAICc < 2 criterion, revealed substantial spatial heterogeneity in the relationships between endemic richness and environmental predictors. Local model performance varied widely across Türkiye, with a mean local *R* ^2^ of 57%, higher than the global pseudo‐*R* ^2^ (50%), indicating marked spatial variation in explanatory power. The highest local *R* ^2^ values occurred in southern Anatolia, particularly along the Central Taurus Mountains, the most endemic‐rich region, where model fit often exceeded 70% (Figure 5).

## 4 Discussion

Our study provides the first nationwide, spatially explicit analytical framework for understanding how species and endemic richness in Brassicaceae, a large, ecologically and evolutionarily diverse plant family (Franzke et al. 2011), are structured across the topographically complex landscapes of Anatolia, thereby bridging a major gap in biogeographical knowledge for one of the world's plant‐diversity “dark spots” (Ondo et al. 2024). Our results show that species and endemic richness peak in southern Anatolia (the Western and Central Taurus Mountains) and along the Anatolian Diagonal, and that these patterns are shaped predominantly by environmental gradients rather than by purely spatial structure. Notably, Brassicaceae includes a high proportion of endemic species in Türkiye (~41%), underscoring the biogeographic and conservation relevance of Anatolia at the intersection of three global biodiversity hotspots. Below, we interpret these results in relation to Anatolia's physiography and environmental gradients.

### 4.1 Sampling Bias and Robustness of Richness Patterns

As in most biogeographical studies based on herbarium data, our inferences are potentially affected by non‐uniform sampling effort (Yang et al. 2013; Meyer et al. 2016; Daru et al. 2018). Collecting intensity in Türkiye has historically been higher in accessible and biologically attractive regions (e.g., the Western and Central Taurus Mountains) and around major urban centers, whereas several remote or politically sensitive areas, such as the Southeastern Taurus (e.g., Hakkari) Mountains and the Northern Zagros Mountains, remain comparatively under‐sampled (Davis 1965–1985; Davis et al. 1988; Güner et al. 2000; Şenkul and Kaya 2017; Figure S1). Nevertheless, several lines of evidence indicate that the richness patterns and statistical relationships recovered here represent genuine biological signals rather than mere artifacts of sampling bias. First, the Brassicaceae hotspots we identify closely coincide with well‐established centers of biodiversity and endemism in Anatolia, particularly along the Western and Central Taurus Mountains and the Anatolian Diagonal, documented in independent floristic syntheses and checklists (Davis 1965–1985, 1971; Davis et al. 1988; Ekim and Güner 1986; Medail and Quezel 1997; Güner et al. 2000, 2012; Şenkul and Kaya 2017; Noroozi et al. 2019; Parolly 2020; IUCN 2025). Second, both species and endemic richness exhibit broad, spatially coherent gradients rather than isolated collector hotspots, and these gradients remain robust across alternative definitions of spatial neighborhoods, multiple modeling approaches (OLS, SAR, SEM, and SAC), and different candidate predictor sets. Third, environmental predictors explain a substantial portion of the variation in both species and endemic richness even after accounting for spatial structure, whereas the spatial‐only fraction is negligible for both components of richness. Notably, the fact that local model performance peaks in the Central Taurus Mountains, one of the regions with the highest overall species richness and endemism, provides additional support against sampling bias. Together, these lines of evidence reinforce the conclusion that the Brassicaceae richness patterns reflect underlying biodiversity patterns across Anatolia rather than sampling artifacts.

### 4.2 Species Richness

Species richness peaks in southern Anatolia, particularly along the Western and Central Taurus Mountains, and extends northeastward through the Anatolian Diagonal, with additional peaks in the northern parts of the Central Anatolian Plateau. Across the best‐supported and parsimonious spatial models, topographic roughness has a strong positive effect, whereas annual precipitation (BIO12) has a strong negative effect. Accordingly, species richness is highest in topographically heterogeneous, relatively dry landscapes. Although environmental predictors explain a moderate portion (34%) of the variation at the national scale, their effects are not spatially uniform: the strongest relationships (> 55%) occur in the Central Taurus Mountains, an area that also corresponds to one of the main centers of overall species richness in Anatolia (see Section 4 above) and to one of the 52 putative refugia identified for the Mediterranean Basin (Médail and Diadema 2009). This spatial congruence indicates that the processes driving species richness are most strongly manifested in this region. Together, these results align with global findings for the positive effect of topographic heterogeneity on species richness, primarily by enhancing habitat diversity and steep environmental gradients (Stein et al. 2014; Antonelli et al. 2018; Rahbek et al. 2019). Although the negative effect of water availability on species richness departs from the positive water–energy relationships typically reported at broad scales (Currie 1991; Hawkins et al. 2003; Kreft and Jetz 2007), it is fully consistent with the ecological affinities of Brassicaceae, which in Anatolia include a strong Irano‐Turanian element, for dry, open habitats (Franzke et al. 2011). In Anatolia, much of the family's diversity belongs to highland elements associated with montane steppe and rocky substrates (Mohammadin et al. 2017), whereas wetter, more forested regions provide fewer suitable niches and may impose stronger competitive exclusion. As a result, species richness declines toward wetter areas. In this sense, our results provide quantitative support for Davis's early view of the Anatolian flora as a system in which “aridity as an evolutionary stimulus” (Stebbins Jr 1952) and eco‐geographical isolation, mediated by steep and dissected topography, jointly promote rapid diversification (Davis 1971).

### 4.3 Endemic Richness

Like species richness, endemic richness peaks in southern Anatolia, particularly along the Western and Central Taurus Mountains, and extends northeastward through the Anatolian Diagonal, reinforcing the central role of these regions as a joint center of diversity and endemism for Brassicaceae (e.g., Mohammadin et al. 2017). However, the environmental drivers of endemic richness are more complex, reflecting a sensitivity to multiple dimensions, including elevational, thermal, hydric, and long‐term climatic stability gradients. This pattern is expected for range‐restricted taxa, which typically occupy narrower, more specialized niches and are therefore more strictly limited by the interplay of topography, climate, and long‐term climatic stability than widespread species (Jansson 2003; Sandel et al. 2011; Slatyer et al. 2013). Across the best‐supported and parsimonious spatial models, elevation and topographic roughness have strong positive effects, whereas mean annual temperature (BIO1), past‐climatic stability index (CSI and CSI‐BIO1), and aridity index (AI) have strong negative effects. Accordingly, endemic richness is highest in high‐elevation, topographically heterogeneous, cooler, climatically stable, and relatively dry landscapes. As in species richness, the strongest relationships occur in the Central Taurus Mountains, an area that also corresponds to one of the main centers of overall endemism in Anatolia (see Section 4 above) and to one of the 52 putative refugia identified for the Mediterranean Basin (Médail and Diadema 2009). This overlap underscores this region as the primary area where the processes driving endemic richness are strongest. These results align well with global findings showing that narrow‐ranged and endemic species concentrate in topographically heterogeneous, cooler, climatically stable montane regions, where rugged terrain, steep environmental gradients, and long‐term persistence in refugial areas promote both speciation (through isolation) and persistence (through stability) (Medail and Quezel 1997; Hobohm 2014; Harrison and Noss 2017; Aykurt et al. 2025). Consistent with this pattern, endemic species exhibit a stronger dependence on high‐elevation and historically stable environments than the overall Brassicaceae flora of Türkiye, indicating that endemic species are more tightly constrained by current topography and the legacies of Pliocene–Quaternary climatic dynamics. This specific combination of drivers suggests that these montane systems function simultaneously as a “museum” preserving ancient lineages in stable refugia and a “cradle” generating new diversity through isolation in rugged terrains, that is, through topographically mediated eco‐geographical isolation (Davis 1971).

### 4.4 Geological History and Diversification in Anatolia

Our results suggest that the Brassicaceae richness patterns we document are tightly linked to Anatolia's geological history (as outlined in Figure 1). The convergence of distinct continental fragments and multi‐phase uplift (Şengör and Yilmaz 1981; Okay and Tüysüz 1999; Okay 2008; Okay et al. 2020) has generated high, topographically and climatically complex, and edaphically heterogeneous landscapes in which Brassicaceae diversity is concentrated in southern Anatolia and along the Anatolian Diagonal. Although the Anatolian Diagonal has often been described as a biogeographical barrier for many taxa (Davis 1971; Ekim and Güner 1986; Gür 2016; Dumlupınar et al. 2023), our family‐level results suggest that, for Brassicaceae, this mountain chain also functions as a high‐altitude biodiversity corridor, facilitating connectivity between the Taurus and Pontic systems. Notably, the Central Taurus Mountains, forming a key segment of the Tauride orogenic belt along the southern margin of the Anatolide–Tauride Block (see Figure 1), represent a nexus of high species richness and endemism, strong topographic and climatic controls, and long‐term refugial stability (Médail and Diadema 2009), coinciding with geochronological evidence for particularly rapid late Quaternary uplift (Öğretmen et al. 2018; Okay et al. 2020). This recent rapid uplift suggests that the region has acted as an active “speciation pump”, where tectonic fragmentation accelerated lineage divergence in the recent geological past.

### 4.5 Limitations and Future Directions

Despite the breadth of our dataset and the use of a spatially explicit analytical framework, several limitations should be acknowledged. First, our analyses are conducted at a relatively coarse resolution (0.5° × 0.5°), which is appropriate for national‐scale biogeographical inference, but less suitable for fine‐grained ecological niche modeling approaches, as it inevitably obscures fine‐scale variation, microrefugia, and local habitat heterogeneity. Second, while we demonstrated the robustness of our richness patterns against sampling bias, we did not explicitly include sampling effort as a covariate in the spatial models. Third, we were unable to incorporate direct measures of tectonics and substrate (e.g., uplift rates, bedrock type, and edaphic properties) into our spatial models. Consequently, geological effects are captured only via indirect proxies (elevation, topographic roughness, and broad climatic indices), which are informative, but insufficient for explicitly testing substrate‐ or age‐related hypotheses. Future studies should therefore build upon Figure 1 by integrating spatial layers of uplift rate, bedrock geology, and edaphic properties into the modeling framework. Fourth, our long‐term climatic‐stability indices focus on Pliocene–Quaternary dynamics and do not explicitly encode older Neogene or Paleogene events. Finally, our analysis focused on contemporary environmental drivers and did not incorporate phylogenetic data, precluding explicit tests of evolutionary hypotheses. These limitations do not undermine the main environmental patterns we report, but highlight important axes for future research.

## 5 Conclusions

We demonstrate that both species and endemic richness peak in southern Anatolia and along the Anatolian Diagonal, confirming these regions as critical centers of diversification and persistence for the Anatolian flora. At the same time, the distinct environmental fingerprints of species and endemic richness underscore that not all components of diversity respond to the same drivers: endemic richness is more tightly coupled to elevation and long‐term climatic stability than species richness. These insights have direct conservation implications. In a country that already ranks among the world's plant‐diversity “dark spots,” maintaining the integrity of topographically complex, climatically stable mountain systems in southern Anatolia and along the Anatolian Diagonal should be treated as a priority for safeguarding both current diversity and the evolutionary potential of the Anatolian flora. By combining a comprehensive taxonomic and occurrence dataset with a spatially explicit analytical framework, our study establishes a biogeographical baseline that can be extended to other plant groups and integrated with tectonic, edaphic, phylogenetic, and functional trait information to further elucidate how Anatolia's unique geological history continues to shape its biodiversity.

## Author Contributions

**İlayda Dumlupınar:** conceptualization (equal), data curation (equal), formal analysis (equal), methodology (equal), visualization (equal), writing – review and editing (equal). **Barış Özüdoğru:** conceptualization (equal), data curation (equal), funding acquisition (lead), methodology (equal), supervision (equal), visualization (equal), writing – review and editing (equal). **Hakan Gür:** conceptualization (equal), formal analysis (equal), methodology (equal), supervision (equal), visualization (equal), writing – original draft (lead), writing – review and editing (equal).

## Funding

This work was supported by the Scientific Research Projects Coordination Unit of Hacettepe University (FHD‐2023‐20678).

## Conflicts of Interest

The authors declare no conflicts of interest.

## Supporting information


**Data S1:** ece373246‐sup‐0001‐Supinfo.docx.

## Data Availability

The data that support the findings of this study are openly available in Zenodo at https://doi.org/10.5281/zenodo.17867065.
