# Supplementary material for: A Plant‐Diversity Dark Spot at the Intersection of Three Biodiversity Hotspots: Environmental Drivers of Brassicaceae Richness in Türkiye
Source: Ecol Evol. 2026 Mar 16;16(3):e73246. doi: 10.1002/ece3.73246 (PMC13093517; doi:10.1002/ece3.73246)
Supplement: Supplementary file 1 — Data S1: ece373246‐sup‐0001‐Supinfo.docx. [file ECE3-16-e73246-s001.docx]

**SUPPORTING INFORMATION**

**Figure S1.** Georeferenced occurrence records of Brassicaceae in Türkiye after data cleaning, plotted on a grid of 0.5° × 0.5° cells used for richness analyses. Red points show unique records (n = 15,547), and black lines delineate the grid cells. For qualitative comparison, the nationwide dataset of 9,677 locations for 2,900 endemic vascular plant taxa in Türkiye (Şenkul and Kaya, 2017) shows a broadly similar large‐scale spatial distribution of records, suggesting that overall sampling coverage for Brassicaceae is comparable to that for the endemic flora, even though the latter includes only endemic taxa.


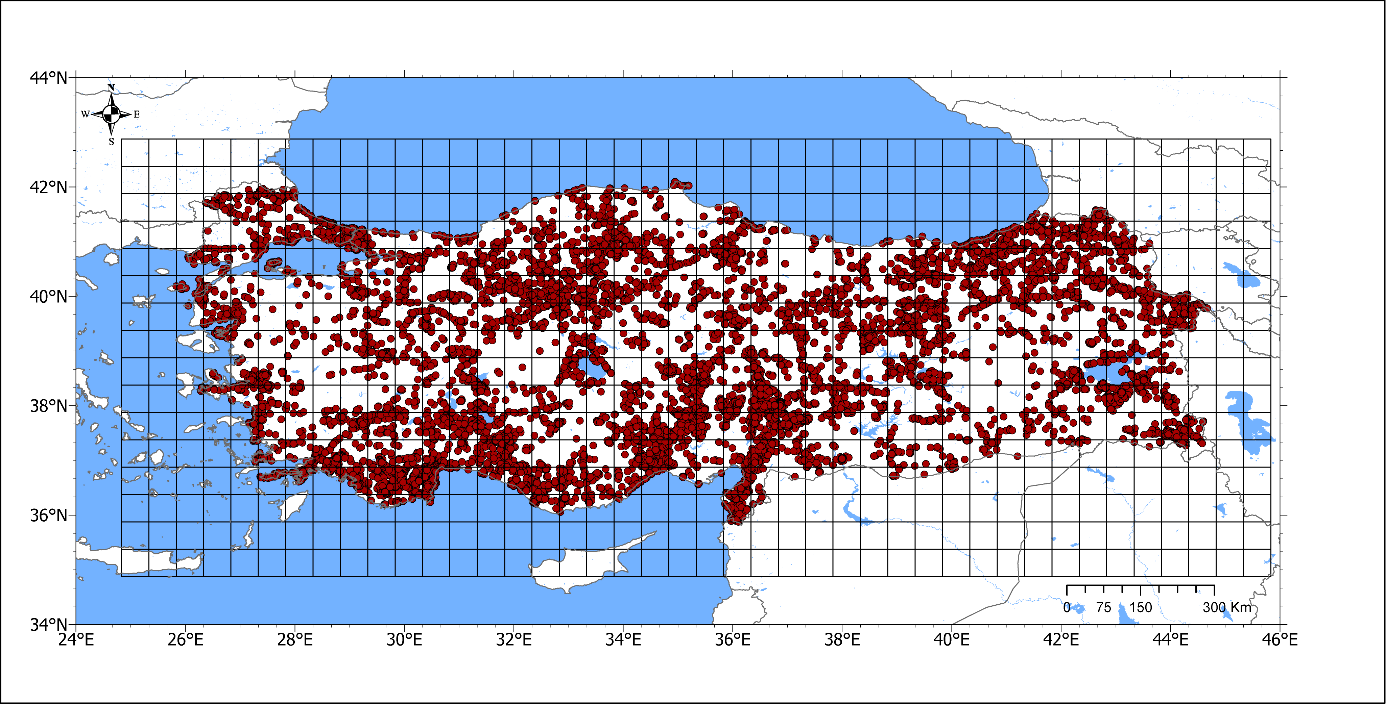


**Figure S2.** Spatial distribution of environmental predictors across Türkiye. BIO1, Mean annual temperature. BIO4, Temperature seasonality. BIO12, Annual precipitation. BIO15, Precipitation seasonality. AI, Aridity index. CSI, General past-climatic stability index. CSI-BIO1, Past-climatic stability index for mean annual temperature. CSI-BIO12, Past-climatic stability index for annual precipitation. NPP, Net primary production. HM, Human Modification.

**
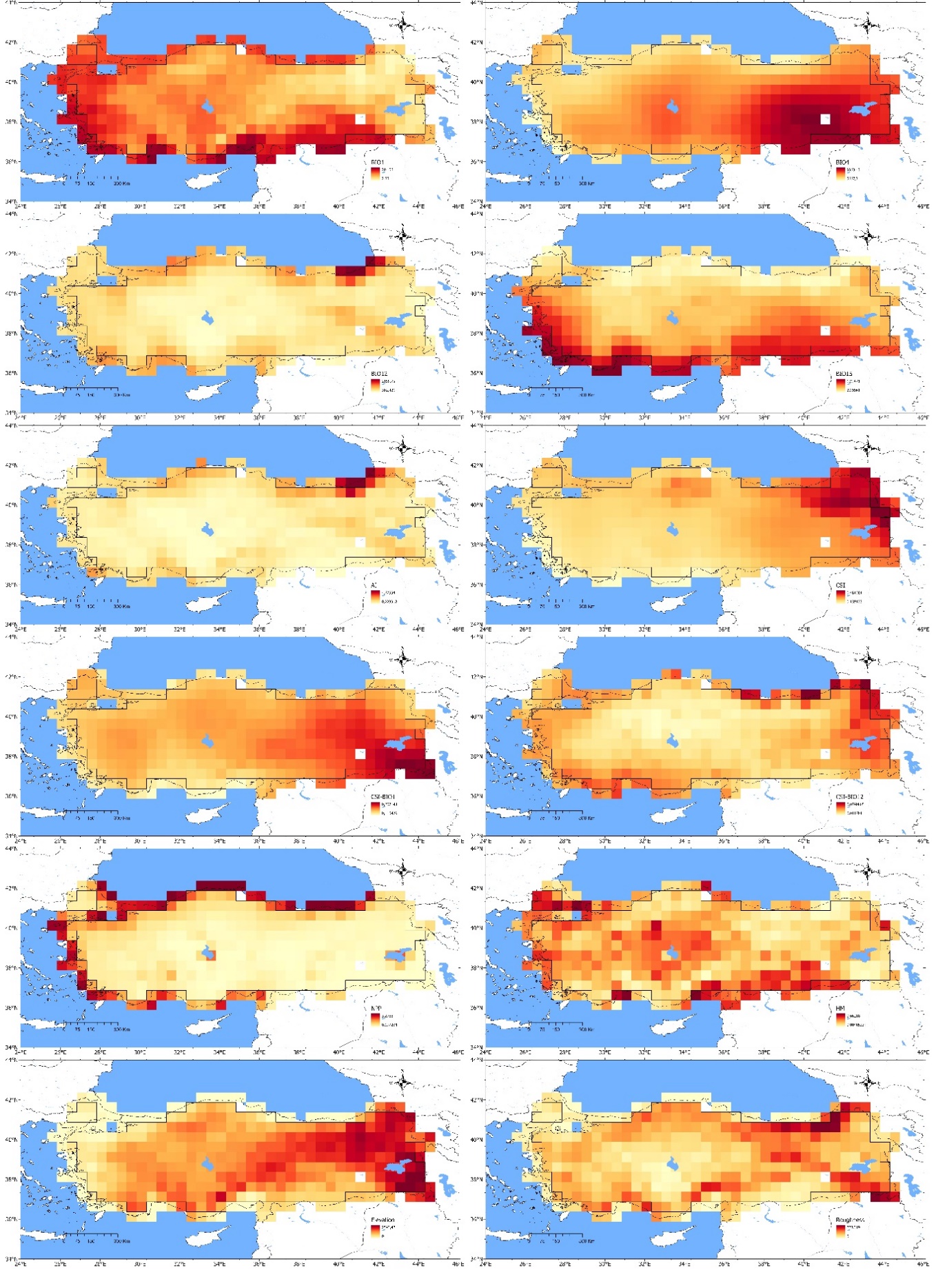
**

**Figure S3.** Bivariate scatterplots of Brassicaceae species richness in Türkiye against environmental predictors (BIO1, BIO4, BIO12, BIO15, AI, CSI, CSI-BIO1, CSI-BIO12, NPP, HM, elevation, and roughness). Each panel shows the relationship between untransformed species richness and a single untransformed predictor, used for exploratory inspection of overall distributional patterns and data structure prior to transformation and modelling. The solid line is the ordinary least-squares (OLS) fit, and the shaded envelope denotes the 95% confidence interval. BIO1, Mean annual temperature. BIO4, Temperature seasonality. BIO12, Annual precipitation. BIO15, Precipitation seasonality. AI, Aridity index. CSI, General past-climatic stability index. CSI-BIO1, Past-climatic stability index for mean annual temperature. CSI-BIO12, Past-climatic stability index for annual precipitation. NPP, Net primary production. HM, Human Modification.

**Figure S4.** Bivariate scatterplots of Brassicaceae endemic richness in Türkiye against environmental predictors (BIO1, BIO4, BIO12, BIO15, AI, CSI, CSI-BIO1, CSI-BIO12, NPP, HM, elevation, and roughness). Each panel shows the relationship between untransformed endemic richness and a single untransformed predictor, used for exploratory inspection of overall distributional patterns and data structure prior to transformation and modelling. The solid line is the ordinary least-squares (OLS) fit, and the shaded envelope denotes the 95% confidence interval. BIO1, Mean annual temperature. BIO4, Temperature seasonality. BIO12, Annual precipitation. BIO15, Precipitation seasonality. AI, Aridity index. CSI, General past-climatic stability index. CSI-BIO1, Past-climatic stability index for mean annual temperature. CSI-BIO12, Past-climatic stability index for annual precipitation. NPP, Net primary production. HM, Human Modification.

**
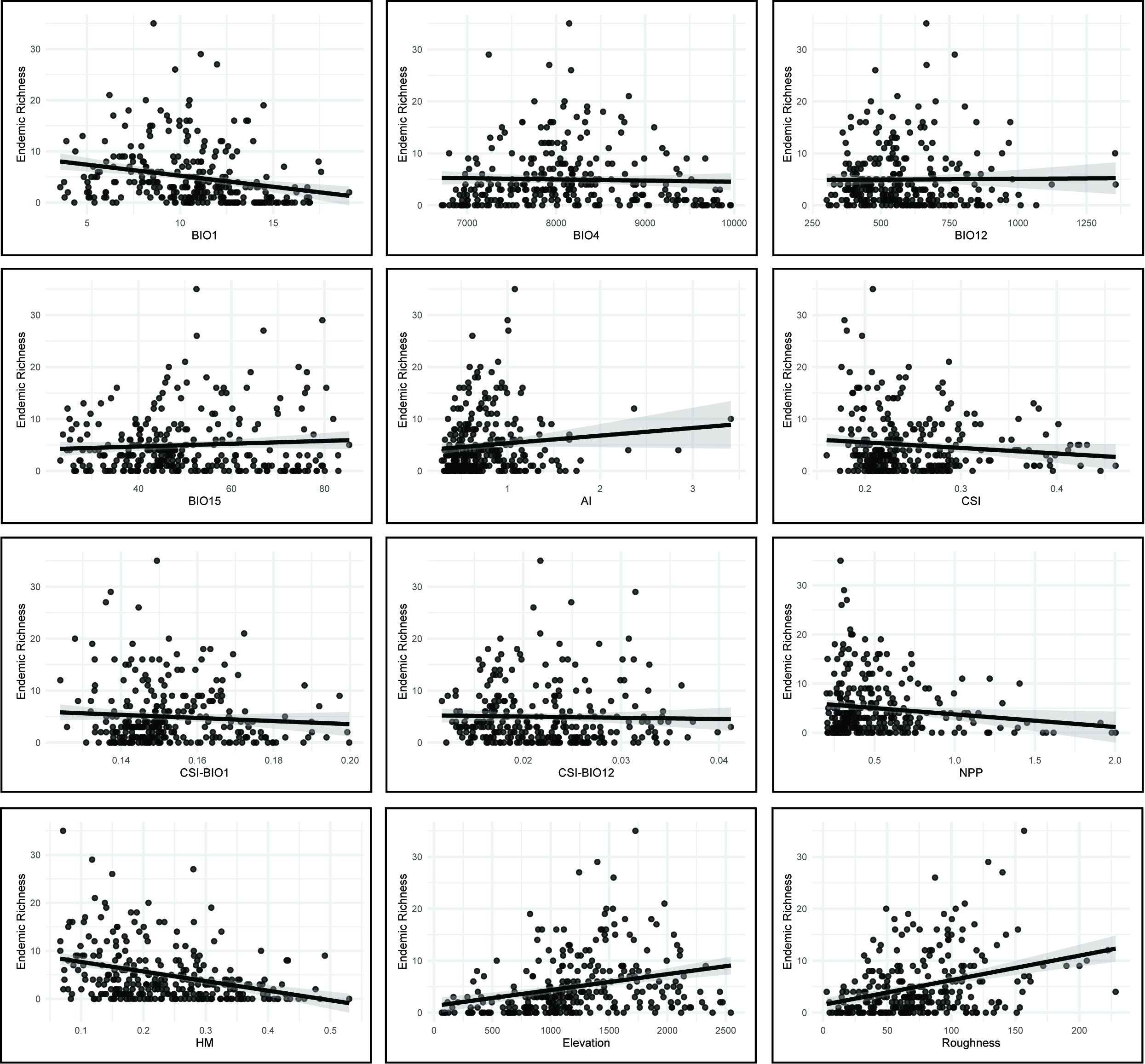
**

**Table S1.** List of herbaria included in this study, with country codes, herbarium acronyms, and numbers of records examined.

| Country Code | Herbarium Code | Herbarium Acronym | Number of Records |
| --- | --- | --- | --- |
| TR | EGE | Ege University Faculty of Science Herbarium | 192 |
| TR | NGBB | Nezahat Gökyiğit Botanical Garden Hebarium | 231 |
| DE | B | Berlin Botanical Garden and Botanical Museum | 329 |
| TR | AKDU | Akdeniz University Herbarium | 416 |
| AT | W | Natural History Museum Vienna | 771 |
| TR | GAZI | Gazi University Herbarium | 1759 |
| TR | ISTE | İstanbul University Faculty of Pharmacy Herbarium | 2828 |
| TR | HUB | Hacettepe University Herbarium | 2866 |
| TR | ANK | Ankara University Faculty of Science Herbarium | 3140 |
| GB | E | Royal Botanic Garden, Edinburgh | 4157 |

**Table S2.** Variables used in this study, with abbreviations, names, units, time ranges, and data sources.

| Variable Abbreviation | Variable Name | Unit | Source |
| --- | --- | --- | --- |
| BIO1 | Annual Mean Temperature | °C*10 | Chelsa v2.1  (1981 - 2010)  <https://www.chelsa-climate.org/>  (Karger et al., 2017) |
| BIO4 | Temperature Seasonality | Standard Deviation*100 | Chelsa v2.1  (1981 - 2010)  <https://www.chelsa-climate.org/>  (Karger et al., 2017) |
| BIO12 | Annual Precipitation | mm/year | Chelsa v2.1  (1981 - 2010)  <https://www.chelsa-climate.org/>  (Karger et al., 2017) |
| BIO15 | Precipitation Seasonality | Coefficient of Variation*100 | Chelsa v2.1  (1981 - 2010)  <https://www.chelsa-climate.org/>  (Karger et al., 2017) |
| AI | Aridity Index | Dimensionless | Chelsa v2.1  (1981 - 2010)  <https://www.chelsa-climate.org/>  (Karger et al., 2017) |
| CSI | Past-climatic stability index | Standard Deviation | Climate Stability Index  M2 (3.3 Ma, Pliocene) - Anthropocene (1979–2013)  <https://doi.org/10.6084/m9.figshare.14672637>  (Herrando-Moraira et al., 2022) |
| CSI-BIO1 | Past-climatic stability index for mean annual temperature | °C | Climate Stability Index  M2 (3.3 Ma, Pliocene) - Anthropocene (1979–2013)  <https://doi.org/10.6084/m9.figshare.14672637>  (Herrando-Moraira et al., 2022) |
| CSI-BIO12 | Past-climatic stability index for annual precipitation | mm | Climate Stability Index  M2 (3.3 Ma, Pliocene) - Anthropocene (1979–2013)  <https://doi.org/10.6084/m9.figshare.14672637>  (Herrando-Moraira et al., 2022) |
| NPP | Net Primary Productivity | kg C/m^2^/year | MODIS/Terra Net Primary Production Yearly L4 Global 500m SIN Grid V006  (2001-2023)  <https://doi.org/10.5067/MODIS/MOD17A3H.006> |
| HM | Human Modification | Dimensionless | Human Modification v3  (2020) <https://hm-30x30.projects.earthengine.app/view/hm-v3>  (Theobald et al., 2025) |
| Roughness | Topographic Roughness | Meters | EarthEnv  <https://www.earthenv.org>  (Amatulli et al., 2018) |
| Elevation | Elevation | Meters | EarthEnv  <https://www.earthenv.org>  (Amatulli et al., 2018) |
